# Ironing Out Possible Micronutrient Deficiencies Associated with Incretin Receptor Agonist-Based Therapies: Proposed Practical Strategies to Prevent and Manage Iron Deficiency

**DOI:** 10.3390/nu18132038

**Published:** 2026-06-23

**Authors:** Marco Infante, Camillo Ricordi, Francesca Pacifici, Donatella Pastore, Raffaele Infante, Massimiliano Caprio, Francesca Chiereghin, Alessandro De Stefano, Giulia Frank, Alessio De Rose, Lorenzo Romano, Laura Di Renzo, Valentina Rovella, Antonino De Lorenzo, Giulia Donadel, David Della-Morte

**Affiliations:** 1 Section of Diabetes & Metabolic Disorders, Faculty of Medicine and Surgery, UniCamillus-Saint Camillus International University of Health Sciences, Via di Sant’Alessandro 8, 00131 Rome, Italy; 2 Division of Cellular Transplantation, Department of Surgery, Cell Transplant Center, Diabetes Research Institute (DRI), University of Miami Miller School of Medicine, 1450 NW 10th Ave., Miami, FL 33136, USA; 3 Section of Food Chemistry, Clinical Nutrition and Pharmaceutical Science, Department of Biomedicine and Prevention, University of Rome Tor Vergata, Via Montpellier 1, 00133 Rome, Italy; 4 Department for the Promotion of Human Sciences and Quality of Life, San Raffaele Roma Open University, Via di Val Cannuta 247, 00166 Rome, Italy; 5 Laboratory of Cardiovascular Endocrinology, IRCCS San Raffaele Roma, Via di Val Cannuta 247, 00166 Rome, Italy; 6 Department of Clinical Sciences and Translational Medicine, University of Rome Tor Vergata, Via Montpellier 1, 00133 Rome, Italy; 7 Hepatology, Clinical Nutrition and Geriatrics Unit, Policlinico Tor Vergata (PTV) Hospital, University of Rome Tor Vergata, Viale Oxford 81, 00133 Rome, Italy; 8 Department of Systems Medicine, University of Rome Tor Vergata, Via Montpellier 1, 00133 Rome, Italy; 9 IRCCS San Raffaele Roma, Via di Val Cannuta 247, 00166 Rome, Italy; 10 Department of Biomedicine, Catholic University “Our Lady of Good Counsel”, Rruga Dritan Hoxha, 1001 Tirana, Albania; 11 Department of Neurology, Evelyn F. McKnight Brain Institute, University of Miami Miller School of Medicine, 1120 NW 14th Street, Miami, FL 33136, USA

**Keywords:** incretin receptor agonists, GLP-1 RA, dual GIP/GLP-1 RA, iron homeostasis, iron deficiency, iron deficiency anemia, IDA, type 2 diabetes, overweight, obesity

## Abstract

Over the last years, incretin receptor agonists—including glucagon-like peptide-1 (GLP-1) receptor agonists (GLP-1 RA) and the dual glucose-dependent insulinotropic polypeptide (GIP)/GLP-1 receptor agonist tirzepatide—have dramatically improved the management of type 2 diabetes, overweight and obesity. However, as the use of incretin receptor agonists continues to increase worldwide, micronutrient deficiencies—including iron deficiency—have emerged as newly recognized adverse effects of these drugs. The present article aims to discuss recent preliminary observational evidence on the potential relationship between incretin receptor agonist-based therapies and the development of iron deficiency and iron deficiency anemia (IDA), as well as the potential mechanisms by which incretin receptor agonists may affect iron homeostasis. Potential mechanisms and factors underlying the development of iron deficiency and IDA in patients treated with incretin receptor agonist-based therapies include inadequate dietary iron intake (due to incretin receptor agonist-mediated reduction in food intake and/or gastrointestinal adverse effects of incretin receptor agonists), low dietary variety, monotonous diets, and changes in food preferences, as well as impairment of intestinal iron absorption (due to delayed gastric emptying, reduced small intestinal motility and/or decreased gastric acid secretion caused by incretin receptor agonists). Moreover, vitamin B2 (riboflavin) deficiency and changes in gut microbiota composition are hypothetical mechanisms that may partly explain iron deficiency in patients treated with incretin receptor agonists, although these hypotheses require confirmation through mechanistic studies. Even though iron deficiency and IDA currently appear to be uncommon adverse effects of incretin receptor agonist-based therapies, clinicians should be aware of the possibility of their occurrence to ensure appropriate prevention and management of these nutritional complications. Nevertheless, future prospective studies are certainly needed to better establish the causal relationship between the initiation of incretin receptor agonist-based therapies and the development of iron deficiency/IDA, as well as the exact mechanisms underlying the potential development of these nutritional complications in patients treated with incretin receptor agonists. Meanwhile, the prescription of incretin receptor agonists should not be unjustifiably restricted by the possible and modest risk of iron deficiency and IDA in patients with one or more approved indications for therapeutic use of these agents. Since no established guidelines currently exist for the prevention and management of iron deficiency and IDA in patients treated with incretin receptor agonists, we herein propose practical strategies to address these possible nutritional complications of incretin receptor agonist-based therapies. These proposed strategies should only be regarded as practical clinical approaches deriving from the existing recommendations for the prevention and management of iron deficiency and IDA, although their cost-effectiveness for the prevention and management of incretin receptor agonist-associated iron deficiency/IDA should be appropriately assessed in future clinical trials.

## 1. Introduction: Incretin Receptor Agonists and Micronutrient Deficiencies

Incretin receptor agonists are drugs that mimic the physiologic actions of gut-derived hormones (also referred to as “incretins”) secreted from enteroendocrine cells in response to food intake [1,2,3]. This drug class includes glucagon-like peptide-1 (GLP-1) receptor agonists (GLP-1 RA) and the dual glucose-dependent insulinotropic polypeptide (GIP)/GLP-1 receptor agonist tirzepatide [2]. GLP-1 RA and the dual GIP/GLP-1 receptor agonist tirzepatide act by reducing appetite, increasing satiety, slowing gastric emptying, enhancing glucose-dependent insulin secretion from pancreatic beta cells and regulating glucagon secretion from pancreatic alpha cells [4,5]. Over the last years, incretin receptor agonists have dramatically improved the management of type 2 diabetes (T2D), overweight and obesity [2]. Furthermore, incretin receptor agonists have been shown to confer cardiorenal protection [6,7,8,9] and to exert significant beneficial effects in patients with metabolic dysfunction-associated steatohepatitis (MASH) and obstructive sleep apnea (OSA) [10,11]. Indeed, incretin receptor agonists have recently been approved for additional indications besides the treatment of T2D and overweight/obesity, namely: reduction in cardiovascular risk in adult patients with overweight/obesity and/or T2D [12,13]; reduction in the risk of worsening kidney disease, kidney failure, and death related to cardiovascular disease (CVD) in adult patients with T2D and chronic kidney disease (CKD) [14]; treatment of MASH in adults [15]; treatment of moderate-to-severe OSA in adults with obesity [16]. Remarkably, the beneficial pleiotropic effects of incretin receptor agonists are likely mediated, at least in part, by the ability of these drugs to reduce systemic inflammation and oxidative stress [17,18].

As the use of incretin receptor agonists continues to increase worldwide [19,20,21], newly recognized adverse effects of these drugs—beyond their known gastrointestinal side effects [22]—are emerging, including micronutrient deficiencies [23,24,25]. These micronutrient deficiencies (particularly deficiencies of vitamin D, B-complex vitamins and iron) appear to be related, at least in part, to appetite suppression, reduced food intake, delayed gastric emptying and altered nutrient absorption mediated by incretin receptor agonists [23]. The potential risk of iron deficiency associated with incretin receptor agonist-based therapies is clinically relevant since iron is a key micronutrient essential for the production and function of hemoglobin and myoglobin, as well as for other cellular processes, such as neurotransmitter synthesis and mitochondrial energy production [26,27].

The present article aims to discuss recent preliminary observational evidence on the potential relationship between incretin receptor agonist-based therapies and iron deficiency/iron deficiency anemia (IDA)/decreased hemoglobin levels [28,29], as well as the potential mechanisms by which incretin receptor agonists may affect iron homeostasis. In this article, we also propose practical strategies for the prevention and management of iron deficiency and IDA in patients treated with incretin receptor agonists.

The present article was conceived as a perspective article based on the recent findings reported by Scott Butsch et al. [28]. Then, a literature search was conducted in the PubMed/MEDLINE and Scopus databases from inception to 26 May 2026 using the following keyword combinations: “GLP-1” or “GLP-1 receptor agonist” or “GLP-1 RA” or “Glucagon-like peptide-1” or “Glucagon-like peptide-1 receptor agonist” or “GIP” or “GIP receptor agonist” or “GIP RA” or “Glucose-dependent insulinotropic polypeptide” or “Glucose-dependent insulinotropic polypeptide receptor agonist” or “dual GIP/GLP-1 RA” or “dual GIP/GLP-1 receptor agonist” AND “iron” or “iron deficiency” or “iron deficiency anemia” or “iron deficiency anaemia” or “IDA” or “anemia” or “anaemia”. Based on the aforementioned literature search, relevant clinical studies related to the topic of this perspective article were identified and discussed. This article did not discuss pregnancy as one of the most common causes of iron deficiency (due to the pregnancy-related increase in iron requirements) [26], since incretin receptor agonists are currently not approved for use in pregnant women [30].

## 2. Incretin Receptor Agonist-Based Therapies, Iron Deficiency and Iron Deficiency Anemia (IDA)

Absolute iron deficiency is a state of low total body iron stores [31]—typically defined as serum ferritin lower than 30 ng/mL or transferrin saturation (TSAT) lower than 20% [calculated as (serum iron/total iron binding capacity) × 100]—and affects nearly 2 billion people worldwide [26]. Indeed, ferritin is the primary form of stored iron, and serum ferritin represents an established marker of total body iron stores [26,32]. Iron deficiency anemia (IDA)—defined as serum ferritin lower than 30 ng/mL or TSAT lower than 20%, and hemoglobin values below the reference range (<12 g/dL in women and <13 g/dL in men)—represents the most common form of anemia worldwide, affecting more than 1.2 billion people on a global scale [26,33,34]. Absolute iron deficiency results from increased blood loss, inadequate dietary iron intake, or impaired iron absorption [26].

Functional iron deficiency—identified by normal or elevated serum ferritin levels associated with low TSAT (<20%) [26]—is a state of imbalance between iron demand and iron availability despite apparently adequate total body iron stores due to inflammatory, infectious or malignant diseases [31,35]. In particular, inflammation increases the expression of hepcidin, a peptide hormone synthesized primarily in the liver, which acts by promoting the degradation of the iron exporter ferroportin, thereby preventing intestinal iron absorption and the mobilization of stored iron from cells of the reticuloendothelial system within the liver, spleen and bone marrow [26,36]. This leads to functional iron deficiency and can subsequently result in functional IDA, which is also referred to as anemia of chronic disease (ACD), iron-restricted erythropoiesis, or anemia of inflammation [26].

Iron deficiency and IDA have recently emerged as potential deleterious nutritional consequences of incretin receptor agonist-based therapies [23,28]. In a recent observational, retrospective analysis of de-identified patient-level claims data obtained from 461,382 adults who were newly prescribed GLP-1 RA (between 2017 and 2021) and without a previous diagnosis of nutritional deficiencies, Scott Butsch et al. [28] reported that nutritional deficiencies were diagnosed in 12.7% and 22.4% of patients within 6 months and 12 months after the initiation of GLP-1 RA therapy, respectively. In this study, individuals prescribed GLP-1 RA had a mean age of 52.9 years, were predominantly female (56.3%), and commonly had T2D (80.5%), hypertension (66.3%) and obesity (44.9%). The most frequently prescribed GLP-1 RA were dulaglutide (38.4%), semaglutide (28.8%) and liraglutide (28.4%). Notably, IDA was diagnosed in 1.6% and 3.2% of patients at 6 months and 12 months after the initiation of GLP-1 RA therapy, respectively. Other types of nutritional anemia were diagnosed in 0.5% and 1.1% of patients at 6 months and 12 months after the initiation of GLP-1 RA therapy, respectively. Thus, IDA was the most common type of nutritional anemia observed during GLP-1 RA therapy. However, a comparative analysis of the IDA incidence at 6 and 12 months after the index date in adults with T2D treated with GLP-1 RA and matched adults prescribed metformin only did not reveal statistically significant differences between the two groups: IDA incidence at 6 months, 1.5% vs. 1.4% in metformin-only users and GLP-1 RA users, respectively; IDA incidence at 12 months, 3.0% vs. 2.8% in metformin-only users and GLP-1 RA users, respectively [28].

A retrospective study conducted by Almuammar et al. [29] on a cohort of 700 patients with diabetes and/or obesity who were prescribed GLP-1 RA between March 2021 and October 2022 (for glucose control or for weight loss) found a statistically significant decrease in hemoglobin levels (median decrease: 0.2 g/dL) after GLP-1 RA therapy initiation (median follow-up period: 9 months), with 8.4% of patients (n = 59) developing anemia. Moreover, baseline hemoglobin showed a statistically significant inverse association with anemia development (OR = 0.31, 95% confidence interval [CI]: 0.21–0.44, *p* < 0.01), indicating that higher baseline hemoglobin levels were associated with a lower risk of subsequent occurrence of anemia [29].

Another important finding from the study conducted by Scott Butsch et al. [28] was that nearly 92% of patients had not visited a dietitian 6 months before GLP-1 RA prescription. Identification of any nutritional deficiency was significantly more likely for subjects who visited a dietitian, as measured at 6 months (18.5% versus 12.2%; *p* < 0.01) and at 12 months (29.8% vs. 21.8%; *p* < 0.01) after starting GLP-1 RA therapy [28]. These results underscore the need for routine nutritional screening (before and after the initiation of incretin receptor agonist-based therapies), prompt identification of nutritional deficiencies and the involvement of registered dietitian nutritionists in the management of patients receiving incretin receptor agonist-based therapies [28]. Indeed, nutrition care specialists should ensure that patients properly meet nutritional needs and do not develop nutritional deficiencies before and after the initiation of incretin receptor agonist-based therapies [37,38,39]. This nutritional evaluation should be included in the routine assessment of patients treated with incretin receptor agonists, with particular attention paid to the impact of altered dietary preferences/intakes and common gastrointestinal adverse effects of incretin receptor agonists (especially nausea, dyspepsia, vomiting, constipation, diarrhea) on food consumption and nutrient absorption [37].

## 3. Potential Mechanisms Underlying Iron Deficiency Associated with Incretin Receptor Agonist-Based Therapies

The exact mechanisms by which incretin receptor agonists may predispose patients to iron deficiency and IDA are not fully understood. However, it is likely that multiple mechanisms contribute to iron deficiency and IDA associated with incretin receptor agonist-based therapies. These mechanisms include inadequate dietary iron intake related to reduced food intake, which in turn results from decreased appetite and increased satiety mediated by the actions of incretin receptor agonists on central nervous system regions involved in appetite regulation, as well as by the ability of these drugs to delay gastric emptying and to promote and prolong the sensation of fullness after food ingestion [23,37,40,41,42]. Indeed, appetite suppression and reduced food intake (reduced micro- and macronutrient intake) mediated by incretin receptor agonists can lower energy intake to levels insufficient to meet daily requirements for key micronutrients, including iron [24,43]. The large, rapid reduction in appetite and caloric intake observed with the use of incretin receptor agonists (reductions in total caloric intake ranging from 16% to 39%) can result in insufficient intake of essential vitamins and minerals (including iron) [37,44]. Johnson et al. [45] conducted a cross-sectional study on 69 subjects who had been using GLP-1 RA and the dual GIP/GLP-1 receptor agonist tirzepatide for weight management for at least one month and who completed a 3-day food record to assess micronutrient intake relative to Dietary Reference Intakes (DRIs). Authors found that participants had insufficient intakes of several key nutrients below the DRIs, including iron [45].

Furthermore, lower energy and nutrient intake related to gastrointestinal adverse effects of incretin receptor agonists (especially nausea, dyspepsia, vomiting and diarrhea) can also partly explain the inadequate dietary iron intake in patients treated with these drugs [24,25,43,46]. Indeed, gastrointestinal adverse effects of incretin receptor agonists can temporarily alter habitual eating patterns and typically lead to avoidance of foods that exacerbate these adverse effects [25]. Moreover, gastrointestinal adverse effects of incretin receptor agonists further contribute to reduced food intake (fewer, smaller and less varied meals), thus increasing the risk of nutritional deficiencies (including iron deficiency) [25]. Low dietary variety and monotonous diets are other potential factors underlying the development of iron deficiency and IDA in patients treated with incretin receptor agonists [24,25,43].

Changes in food preferences and reduction in the consumption of iron-rich foods—such as meats, poultry, fish and seafood [43,47]—may also explain the association between the use of incretin receptor agonist-based therapies and the development of iron deficiency and IDA. Indeed, a US survey conducted by Dilley et al. [48] on 1955 current, previous and potential consumers of GLP-1 RA found a decline in the consumption of meat (beef, pork, chicken), fish and seafood among subjects treated with incretin receptor agonists. Further prospective studies are needed to better assess the long-term changes in food consumption patterns caused by incretin receptor agonists, with particular attention to the intake of iron-rich foods.

In addition, delayed gastric emptying and reduced small intestinal motility mediated by incretin receptor agonists [49,50,51] can result in altered timing and efficiency of nutrient delivery to absorption sites in the small intestine, with subsequent impaired absorption of vitamins and minerals (including iron) [25,52]. Since iron is absorbed in the small intestine (mainly in the duodenum and upper jejunum) [47], impairment of intestinal iron absorption appears to be one of the most plausible mechanisms accounting for iron deficiency and IDA in patients treated with incretin receptor agonists. In this regard, Melis et al. [53] conducted a prospective pilot study on 51 adult patients with T2D and poor glucose control who started GLP-1 RA therapy with once-weekly subcutaneous semaglutide. At baseline and at 10 weeks after semaglutide therapy initiation, participants underwent an oral iron absorption test (OIAT) by ingesting a single 350 mg ferrous fumarate capsule (115 mg of elemental iron) following a 12-h fast. Venous blood samples were obtained at baseline and 2 h following the ferrous fumarate capsule ingestion. These samples were analyzed to evaluate blood parameters related to iron metabolism, including serum iron concentration. At 10 weeks following semaglutide therapy initiation, as compared to baseline (before semaglutide therapy initiation), there was a 13% median relative reduction in iron absorption levels. Moreover, 17.6% of the study participants (9 out of 51 participants) experienced at least a 30% decrease in iron absorption with semaglutide therapy as compared to the period before semaglutide therapy initiation [53]. Although based on a small sample size, findings from the study by Melis et al. [53] suggest that the GLP-1 RA semaglutide may reduce intestinal iron absorption and cause iron deficiency by delaying gastric emptying and altering intestinal motility [23,53].

Additionally, incretin receptor agonists may contribute to iron deficiency and IDA by reducing gastric acid secretion. Low gastric pH resulting from physiologic gastric acid secretion is essential for adequate iron absorption [54,55,56,57], since gastric acid promotes iron absorption by favoring reduction and solubilization of dietary ferric iron in the duodenum [54]. It has been shown that GLP-1 inhibits gastric acid secretion via vagal and hormonal mechanisms (increased somatostatin secretion and/or decreased gastrin secretion) [58,59,60,61,62]. Clinical studies previously showed that GLP-1 suppresses the postprandial secretion of gastrin [59] and requires intact vagal innervation of the stomach to exert its inhibitory effect on gastric acid secretion [60]. Conditions or factors associated with absent or reduced gastric acid secretion (achlorhydria/hypochlorhydria) and increased gastric pH (e.g., autoimmune or atrophic gastritis, *Helicobacter pylori* infection, past gastric surgery, older age, use of proton pump inhibitors, histamine H2 receptor antagonists, antacids) [26,55,57,63,64,65,66] may further predispose patients treated with incretin receptor agonists to the development of iron deficiency and IDA.

Further evidence supporting a potential interference of GLP-1 RA with iron absorption and metabolism derives from a study demonstrating that exposure to GLP-1 RA, as compared with absence of GLP-1 RA exposure, was associated with 30% lower circulating ferritin levels and with a 50% longer interval between successive phlebotomies in patients with hereditary hemochromatosis and T2D [67]. These data suggest that GLP-1 RA have the potential to reduce iron stores and circulating ferritin levels. In line with the latter findings, a study conducted in db/db mice—an animal model of T2D [68]—showed that the GLP-1 receptor agonist liraglutide can attenuate hepatic iron overload, oxidative damage and ferroptosis [69]. Moreover, a study conducted on a murine model of hepatic ischemia–reperfusion injury showed that liraglutide alleviates hepatic ischemia–reperfusion injury by decreasing hepatic iron accumulation and suppressing ferroptosis via activation of the GSK3β/Nrf2 and SMAD159/Hepcidin/FTH pathways [70].

Hypothetical mechanisms that may partly explain iron deficiency in patients treated with incretin receptor agonists include vitamin B2 (riboflavin) deficiency and changes in gut microbiota composition. However, mechanistic studies should be conducted to thoroughly investigate and confirm these hypothetical mechanisms.

Patients treated with incretin receptor agonists may develop vitamin B2 deficiency as a consequence of reduced micronutrient intake and/or impaired intestinal micronutrient absorption caused by these drugs [43]. Indeed, vitamin B2 deficiency may reduce iron absorption, increase gastrointestinal iron losses and impair iron mobilization from iron stores [71,72,73,74]. Vitamin B2 deficiency has been reported in patients who have undergone bariatric surgery [75,76]. Therefore, studies are needed to specifically assess dietary vitamin B2 intake, as well as vitamin B2 deficiency and its relationship with iron deficiency in patients treated with incretin receptor agonists. Main dietary sources of vitamin B2 include dairy products, milk, fish, meat, poultry, eggs, nuts, mushrooms, green vegetables, fortified cereals, whole-grain or enriched breads [43,77,78].

Incretin receptor agonists may theoretically alter iron metabolism and absorption even through their ability to modify gut microbiota composition [79,80], which plays an important role in intestinal cells’ iron sensing and in iron homeostasis [81,82]. Future studies are needed to determine whether incretin receptor agonists can induce changes in the abundance of gut bacteria involved in iron absorption and homeostasis, including *Lactobacillus fermentum* [83].

Table 1 lists the potential mechanisms underlying the development of iron deficiency and IDA (beyond classic risk factors for iron deficiency and IDA) in patients treated with incretin receptor agonists.

## 4. Proposed Strategies for the Prevention and Management of Iron Deficiency and IDA in Patients Treated with Incretin Receptor Agonists

A joint Advisory from the American College of Lifestyle Medicine, the American Society for Nutrition, the Obesity Medicine Association and The Obesity Society recently provided evidence-based nutritional and lifestyle strategies to address key challenges of incretin receptor agonist-based therapies in patients with obesity [37]. Among the key nutritional priorities to support the safety and efficacy of incretin receptor agonists for the management of obesity, the Advisory included completion of baseline nutritional assessment and screening (prior to initiation of incretin receptor agonist-based therapies), navigation of dietary preferences and intakes, and the prevention and mitigation of nutrient deficiencies [37]. Nutritional assessment and laboratory testing prior to the initiation of incretin receptor agonist-based therapies are particularly useful in individuals with an established or past nutrient deficiency, a history of a very low-calorie diet, bariatric surgery, celiac disease, or any inflammatory condition predisposing to nutrient deficiencies [37]. Moreover, patients starting incretin receptor agonist-based therapies should regularly receive individualized medical nutrition therapy (provided by registered dietitian nutritionists) according to their specific nutritional needs [37].

In line with existing bariatric care models [84,85,86], laboratory tests aimed to screen for the most common micronutrient deficiencies associated with incretin receptor agonist-based therapies (including iron deficiency) should be performed before and after the initiation of incretin receptor agonists (baseline and periodic biochemical monitoring for micronutrient deficiencies) in subjects at risk for micronutrient deficiencies [28,37,39,43,52]. Currently, there are no established guidelines for the prevention and management of iron deficiency and IDA in patients receiving incretin receptor agonists. Therefore, we herein propose practical strategies for the prevention and management of iron deficiency and IDA in patients treated with incretin receptor agonist-based therapies.

### 4.1. Laboratory Approach to the Prevention and Management of Iron Deficiency and IDA in Patients Treated with Incretin Receptor Agonists

The key biochemical markers of body iron status required for the screening and diagnosis of iron deficiency and IDA are serum ferritin and TSAT [26,87]. Of note, serum ferritin represents the first-line initial test for iron deficiency [26]. Absolute IDA is generally defined by a serum ferritin level below 30 ng/mL, a threshold associated with 92% sensitivity and 98% specificity for absent bone marrow iron stores [26]. However, serum ferritin is not sufficiently reliable as a diagnostic marker of iron deficiency in the presence of overt or subclinical inflammation, including conditions associated with an acute phase response and several common chronic conditions (e.g., heart failure, CKD, inflammatory bowel disease, rheumatic diseases, cancer) [26,88]. In fact, ferritin is an acute phase reactant whose circulating concentrations increase with inflammation [26,89]. In general, serum ferritin levels are rarely greater than 100 ng/mL in patients with absolute iron deficiency and a concomitant inflammatory condition [26]. However, in the presence of inflammatory conditions or an acute phase response, serum ferritin and TSAT should be measured together to accurately determine the presence of iron deficiency and IDA [26]. Based on patients’ signs and symptoms, if iron deficiency is suspected despite a serum ferritin level above 50 ng/mL, TSAT should be evaluated [26]. TSAT measures the percentage of transferrin that is saturated with iron, with low TSAT values (<20%) indicating that there is insufficient bioavailable circulating iron [26]. Low TSAT values are particularly helpful to diagnose iron deficiency and IDA in subjects with chronic diseases (e.g., heart failure, CKD, inflammatory bowel disease, rheumatic diseases, cancer), who may exhibit elevated serum ferritin levels due to inflammation [26]. It is important to outline that TSAT should be measured at least 5 to 9 h after consuming iron-containing foods, multivitamins or supplements, since recent iron intake can lead to apparent increases in serum iron levels [26]. Fasting is not required prior to TSAT measurement if the ingestion of iron-containing foods, multivitamins or supplements is avoided [26].

In order to promptly diagnose and treat iron deficiency or IDA, we suggest performing blood tests aimed to assess complete blood count (CBC; for the assessment of red blood cell [RBC] count, RBC indices [MCV, Mean Corpuscular Volume; MCH, Mean Corpuscular Hemoglobin; MCHC, Mean Corpuscular Hemoglobin Concentration; RDW, Red Blood Cell Distribution Width] and hemoglobin and hematocrit levels) and main biochemical markers of body iron status (serum ferritin, TSAT, serum iron, serum transferrin, total iron-binding capacity [TIBC], unsaturated iron-binding capacity [UIBC]) [87,88,90,91,92,93,94,95] before the initiation of incretin receptor agonist-based therapies in patients with signs and symptoms suggestive of iron deficiency or IDA, in patients with a previously established diagnosis of iron deficiency or IDA, as well as in vulnerable subjects with one or more risk factors for the development or worsening of iron deficiency and IDA (see Section 4.2). In some instances, assessment of reticulocyte hemoglobin content (Ret-Hb) and soluble transferrin receptor (sTfR) may also be useful to detect latent iron deficiency or subtle iron deficiency at the tissue level [96,97].

Overall, blood test results suggesting the presence of absolute IDA include the following: a ferritin level < 30 ng/mL (in the absence of inflammation); TSAT < 20% [26]; low hemoglobin values (<13 g/dL in men; <12 g/dL in nonpregnant women; <11 g/dL in pregnant women) [98]; low MCV values (microcytosis; MCV < 80 fL) [26]; low MCH and/or MCHC values (hypochromia); microcytic hypochromic anemia; reduced serum iron values; elevated TIBC values; elevated serum transferrin values [98,99,100]. Nevertheless, hemoglobin and MCV are poor surrogate markers of iron deficiency and are inadequate for diagnosis, since anemia and microcytosis typically develop only in later stages [26]. Reticulocytosis (defined by a reticulocyte count >100,000/μL) following iron therapy is also considered diagnostic of iron deficiency, since it indicates that previously low iron levels limited erythropoiesis; conversely, absence of reticulocytosis within approximately 1 week after iron therapy suggests alternative or concurrent diagnoses other than iron deficiency [26].

CBC and main biochemical markers of body iron status should be regularly assessed during incretin receptor agonist-based therapies in patients with an established diagnosis of iron deficiency or IDA, as well as in vulnerable subjects with one or more risk factors for the development or worsening of iron deficiency and IDA (see Section 4.2). In the latter subgroups of patients treated with incretin receptor agonists, periodic assessment of CBC and main biochemical markers of body iron status every 3 months may be reasonable. This is consistent with standard clinical practice for the management of patients with iron deficiency or IDA receiving iron supplementation; in these patients, reassessment of CBC and main biochemical markers of body iron status is generally performed 3–6 months after the initiation of iron supplementation [26,90], which represents a retesting interval generally sufficient to allow repletion of iron stores and normalization of serum ferritin levels [90]. Furthermore, this retesting interval for the assessment of CBC and main biochemical markers of body iron status in patients treated with incretin receptor agonists appears reasonable based on the study conducted by Scott Butsch et al. [28], who reported a diagnosis of IDA in 1.6% of patients at 6 months after initiation of GLP-1 RA therapy. If intravenous iron therapy is required, reassessment of CBC and main biochemical markers of body iron status should be performed earlier (4 weeks after intravenous iron infusion), in order to establish whether an additional intravenous iron infusion is needed [26].

Reassessment of CBC and main biochemical markers of body iron status after the initiation of iron supplementation is important to evaluate the need for ongoing iron administration and to establish the optimal dose, dosing frequency and route of administration of iron supplementation [90]. However, the frequency of laboratory workup including CBC and main biochemical markers of body iron status in patients treated with incretin receptor agonists can vary (e.g., closer laboratory monitoring) based on clinical judgement, selected circumstances and/or specific factors (e.g., number of risk factors for the development or worsening of iron deficiency and IDA, occurrence of gastrointestinal adverse effects of incretin receptor agonists during incretin receptor agonist dose up-titration, prolonged gastrointestinal adverse effects of incretin receptor agonists, pronounced appetite suppression, rapid weight loss, development of signs and symptoms suggestive of iron deficiency or IDA, moderate-to-severe IDA).

In patients prescribed incretin receptor agonists who have an established iron deficiency/IDA, a past history of iron deficiency/IDA, an established anemia other than IDA, or a past history of anemia other than IDA, CBC and main biochemical markers of body iron status may be assessed more frequently. In patients prescribed incretin receptor agonist-based therapies with established iron deficiency or IDA, iron supplementation should be started or intensified concomitantly with the initiation of such therapies. Correcting iron deficiency remains essential even in the presence of non-anemic iron deficiency [101] to prevent its progression to IDA, especially in patients treated with incretin receptor agonists, who are at increased risk of developing IDA [28].

### 4.2. Identification of Vulnerable Subjects with Risk Factors for the Development or Worsening of Iron Deficiency and IDA Before the Initiation of Incretin Receptor Agonist-Based Therapies

Among patients eligible for incretin receptor agonist-based therapies, it is important to identify vulnerable subjects with one or more risk factors for the development or worsening of iron deficiency and IDA. Among these patients, low socioeconomic status, female sex, premenopausal status, heavy menstrual bleeding, chronic inflammation, history of bariatric surgery, inadequate iron intake, chronic diseases (including overweight/obesity, T2D, CKD, heart failure, rheumatic diseases and cancer), malabsorption syndromes (such as inflammatory bowel disease and celiac disease), age > 65 years, use of certain medications (e.g., proton pump inhibitors, histamine H2 receptor antagonists, antacids, aspirin, antiplatelet drugs, non-aspirin nonsteroidal anti-inflammatory drugs, oral anticoagulants), vegetarianism and veganism represent major risk factors for the development or worsening of iron deficiency and IDA [26,43,98,102,103,104,105,106,107,108,109,110,111,112,113,114,115,116]. In this context, it is worth highlighting that patients with overweight/obesity and T2D are already prone to anemia due to different factors, such as chronic inflammation, micronutrient deficiencies (including iron deficiency) [43,105,106,107,108,109,117], history of bariatric surgery [118], coexisting CKD [52,119,120,121], and poor glucose control [52,122]. Thus, initiation of incretin receptor agonist-based therapies may increase the vulnerability to anemia and IDA in patients with overweight/obesity and/or T2D. It is also important to note that correction of IDA is desirable in patients with diabetes mellitus or prediabetes, since IDA can cause misinterpretation of glycated hemoglobin (HbA1c) values (falsely elevated HbA1c values) [123].

### 4.3. Recognition of Signs and Symptoms Suggestive of Iron Deficiency or IDA

If the cause of iron deficiency is not diagnosed and appropriately treated, IDA can develop [26]. Absolute iron deficiency gradually progresses from low iron stores to IDA, although subjects with non-anemic iron deficiency or IDA can be asymptomatic [26]. Subjects with IDA may exhibit more severe symptoms than those who have iron deficiency without anemia, particularly if anemia develops quickly (over days or weeks rather than months) [26].

Signs and symptoms suggestive of absolute iron deficiency or IDA should be promptly recognized in patients eligible for incretin receptor agonist-based therapies and in patients who are already receiving incretin receptor agonists, in order to allow appropriate laboratory monitoring and timely intervention. These signs and symptoms (including laboratory findings) include microcytic hypochromic anemia, weakness, fatigue, exercise intolerance, tachycardia, pallor, dyspnea, cold intolerance, koilonychia (spoon-shaped nails), glossitis, cognitive dysfunction, decreased attention and concentration (“brain fog”), mood changes (irritability, depression), lightheadedness, pica (craving non-food substances, such as ice [pagophagia or ice craving]), restless legs syndrome, and worsening heart failure [26,43,124]. Iron deficiency or IDA may also contribute to the fatigue reported as a potential adverse effect of incretin receptor agonist-based therapies [125]. In the presence of signs and symptoms suggestive of iron deficiency or IDA, and upon laboratory confirmation, these nutritional complications of incretin receptor agonists should be promptly managed.

#### Hair Loss: A Potential Consequence of Iron Deficiency During Incretin Receptor Agonist-Based Therapies

Iron deficiency is commonly observed in patients with hair loss, particularly in premenopausal women [126,127,128]. Hair loss has also been reported as a potential adverse effect of GLP-1 RA and dual GIP/GLP-1 RA therapies [129]. However, findings across studies remain conflicting, as cases of hair regrowth have also been reported [129,130]. Although a causal relationship between incretin receptor agonist-based therapies and hair loss cannot be established at this time, the occurrence of hair loss in patients treated with GLP-1 RA or dual GIP/GLP-1 RA should prompt the assessment of CBC and main biochemical markers of body iron status. In fact, iron deficiency is considered one of the reversible nutritional causes of hair loss in patients treated with incretin receptor agonists [43,128,130]. In patients treated with incretin receptor agonists, hair loss can also be due to sustained negative energy balance, insufficient dietary protein and essential amino acid intake, essential fatty acid deficiency, and deficiency of micronutrients other than iron (such as niacin [vitamin B3], biotin [vitamin B7], vitamin D, zinc, selenium) [28,43,130,131].

### 4.4. Dietary Recommendations for the Prevention and Management of Iron Deficiency and IDA in Patients Treated with Incretin Receptor Agonists

According to the aforementioned joint Advisory from the American College of Lifestyle Medicine, the American Society for Nutrition, the Obesity Medicine Association and The Obesity Society [37], dietary recommendations for individuals treated with incretin receptor agonists should prioritize maintaining adequate nutrient intake despite the typically reduced appetite and lower caloric consumption associated with the use of these medications. In order to maintain a good diet quality in the context of decreased caloric intake, clinicians should encourage the consumption of a variety of nutrient-dense, minimally processed foods, including vegetables, fruits, whole grains, legumes, nuts, seeds, and lean proteins (e.g., poultry, fish/seafood) [37]. In fact, nutrient-dense, minimally processed foods represent good dietary sources of vitamins and minerals that may become deficient during incretin receptor agonist-based therapies [37,43]. Patients should also be advised to limit the consumption of refined carbohydrates (e.g., refined grains, flours, starches, and added sugars), sugar-sweetened beverages, red and processed meats, as well as fast foods, savory snacks and ultra-processed sweets [37].

In patients treated with incretin receptor agonists, nutritional consultation should also be aimed at increasing the consumption of selected iron-rich foods, such as lean meat, poultry, fish, shellfish, green leafy vegetables, beans, lentils, peas, nuts, dried fruits, and fortified cereals [43,132]. The two types of iron found in foods are heme iron and non-heme iron. Heme iron is present only in animal-source foods [133]. Meats, poultry, fish and shellfish are animal-source foods high in heme iron [47,133]. On the other hand, plant-based foods (e.g., vegetables, legumes, nuts, seeds, dried fruits, and whole grains) contain exclusively non-heme iron [47,133]. Heme iron is more efficiently absorbed and has a higher bioavailability than non-heme iron [47,133].

Since patients treated with incretin receptor agonists should consume adequate amounts of plant-based foods and non-heme iron from such foods is less well absorbed than heme iron, these patients should also consume adequate amounts of vitamin C-rich foods (particularly citrus fruits, bell peppers, green leafy vegetables, tomatoes, and berries) to enhance the absorption of non-heme iron [43,47,132,134]. Thus, high consumption of vitamin C-rich foods and vitamin C (ascorbic acid) supplementation may be required particularly in subjects following restrictive vegetarian diets [135].

Importantly, many of the nutrient-dense, minimally processed foods recommended for patients treated with incretin receptor agonists are high in fiber, iron and vitamin C [37]. Moreover, it is advisable to avoid the ingestion of calcium supplements, as well as the consumption of tea, coffee, calcium-rich foods and soy products during meals, since these supplements and foods can alter the absorption of non-heme iron [43,47,133,136,137,138].

Notably, many iron-rich foods are also among the protein-rich foods (e.g., poultry, fish/seafood, beans, peas, lentils, nuts, seeds) recommended by the aforementioned joint Advisory to ensure adequate dietary protein intake and preserve muscle and bone mass in individuals treated with incretin receptor agonists [37]. On the other hand, the same joint Advisory recommended minimizing or avoiding the consumption of red and processed meats [37]—despite their high iron content [139]—given its association with a higher risk of cardiovascular disease, T2D and colorectal cancer in the general population [37,140,141,142].

### 4.5. Iron and Vitamin C Supplementation in Patients Treated with Incretin Receptor Agonists

Although adequate dietary iron intake is important for preventing iron deficiency, increased dietary iron intake alone is insufficient to replenish iron stores in patients who have already developed absolute iron deficiency [26]. Patients treated with incretin receptor agonists appear to be at higher risk for micronutrient deficiencies and their related complications (including iron deficiency and IDA) [37,43]. In patients treated with incretin receptor agonists, dietary recommendations alone are likely insufficient to correct micronutrient deficiencies (including iron deficiency), especially in the presence of concomitant conditions or factors that further increase the risk of such adverse effects (e.g., malabsorption syndromes—such as inflammatory bowel disease and celiac disease—or pronounced appetite suppression mediated by incretin receptor agonists) [37,43]. Therefore, dietary supplements (administered at appropriate doses and tailored to each person’s needs) can be considered for patients receiving incretin receptor agonist-based therapies who have established micronutrient deficiencies, including iron deficiency [37,43].

Iron supplements (including iron-containing multivitamins and iron-only preparations) are used particularly when dietary iron intake does not meet nutritional needs [47]. In patients with established iron deficiency or IDA (despite the recommended adequate consumption of foods high in iron and vitamin C) who are prescribed incretin receptor agonists, iron supplementation should be started or intensified before the initiation of incretin receptor agonist-based therapies. Similarly, patients prescribed incretin receptor agonists who develop iron deficiency or IDA after the initiation of incretin receptor agonist-based therapies (despite the recommended adequate consumption of foods high in iron and vitamin C) should promptly start iron supplementation.

In general, the management of iron deficiency or IDA in patients treated with incretin receptor agonists should follow the same principles outlined in the international guidelines for the management of these conditions (particularly in terms of optimal dose, dosing frequency and route of administration of iron supplementation) [26,143,144]. Oral iron represents the first-line treatment for the majority of patients with absolute iron deficiency [26]. When iron supplementation is required in patients receiving incretin receptor agonists, clinicians may prefer newer nanoparticle oral iron formulations with a better tolerability profile (associated with fewer gastrointestinal adverse effects) and improved absorption compared with oral iron salts [145,146,147], given the impaired iron absorption and frequent gastrointestinal adverse effects associated with incretin receptor agonist-based therapies [46,53]. Patients receiving oral iron supplementation should not consume coffee, tea, fiber, or calcium-containing foods within 1 h before or after taking an iron supplement, since these foods can decrease iron absorption [26,98,143,148]. Treatment with oral iron therapy should be continued until ferritin and TSAT (and hemoglobin levels in patients with IDA) have returned to the normal range [26]. If serum ferritin and/or TSAT and hemoglobin levels do not increase despite good adherence to oral iron supplementation, further evaluation is warranted for disorders that impair iron absorption (e.g., autoimmune gastritis or celiac disease), ongoing gastrointestinal bleeding, or alternative causes of anemia [26].

The addition of an oral vitamin C supplement (usually at a dose of 500 mg/day) to oral iron supplementation can be considered to improve iron absorption [143] and to address the possible insufficient dietary intake of vitamin C related to the use of incretin receptor agonists [43,45]. In fact, vitamin C improves iron absorption by exerting a reducing and a chelating effect on iron salts, reducing ferric iron (Fe^3+^) to ferrous iron (Fe^2+^) and preventing the formation of insoluble iron compounds [143,149]. If feasible, taking oral iron with meat protein can improve iron absorption, while vitamin C (at a dose of 500 mg/day) can enhance iron absorption even if dietary fiber or calcium is present in the meal [143].

Intravenous iron therapy may be necessary in the following groups of patients: patients requiring a faster iron replenishment than that achieved with oral iron (e.g., patients with IDA who are scheduled for surgery); patients who have oral iron intolerance; patients in whom oral iron is likely ineffective in the treatment of iron deficiency or IDA (e.g., menstruating women with heavy menstrual bleeding, patients with malabsorption syndromes or impaired iron absorption, patients who underwent bariatric surgery, patients with active inflammatory bowel disease and other chronic inflammatory conditions, patients with chronic gastrointestinal bleeding, patients with ongoing blood loss) [26,150]. The dosing regimen, administration frequency, and duration of intravenous iron therapy vary according to the underlying cause of iron deficiency [26].

### 4.6. Possible Coexistence of Other Nutritional and Non-Nutritional Causes of Anemia in Patients Experiencing Iron Deficiency or IDA During Incretin Receptor Agonist-Based Therapies

If patients prescribed incretin receptor agonist-based therapies continue to exhibit anemia despite dietary recommendations, adequate iron supplementation—with or without combined vitamin C supplementation in the case of oral iron supplementation—and correction of iron deficiency, clinicians should suspect the presence of other (nutritional and/or non-nutritional) causes of anemia. In particular, causes of anemia other than iron deficiency that should be suspected and promptly managed in patients treated with incretin receptor agonists include other types of nutritional anemia, such as anemia related to the deficiency of one or more of the following micronutrients: vitamin B1 (thiamine), vitamin B2 (riboflavin), vitamin B6 (pyridoxine), folic acid (vitamin B9), cobalamin (vitamin B12), vitamin A, vitamin C, and copper [74,151,152,153,154,155,156,157]. In fact, patients treated with incretin receptor agonists may experience a deficiency of one or more of the aforementioned micronutrients [37,39,43,45]. Notably, in patients treated with incretin receptor agonists, iron deficiency may be associated with folic acid deficiency and/or vitamin B12 deficiency [28,37,39,43], the latter occurring more frequently in individuals who are concomitantly treated with metformin and/or with acid-suppressing medications, such as histamine H2 receptor antagonists and proton pump inhibitors [158,159].

### 4.7. Potential Therapeutic Use of SGLT2 Inhibitors to Mitigate the Risk or Improve the Management of Iron Deficiency and IDA in Patients Treated with Incretin Receptor Agonists

In patients with T2D and CKD, who are at increased risk of developing anemia [160], the administration of a sodium-glucose cotransporter 2 (SGLT2) inhibitor in addition to incretin receptor agonist-based therapies may mitigate the risk of IDA associated with incretin receptor agonist-based therapies, while also improving glucose control and providing the cardiorenal benefits related to the use of SGLT2 inhibitors [161,162]. Indeed, SGLT2 inhibitor therapy initiation, as compared to GLP-1 RA therapy initiation, has been associated with a lower incidence of composite anemia outcomes and a lower incidence of anemia events after a median follow-up period of 2.5 years in a cohort of 13,799 patients with T2D and CKD [163]. These findings may be explained by the fact that SGLT2 inhibitors have the potential to improve utilization of iron stores by promoting erythropoietin (EPO) synthesis, stimulating erythropoiesis, increasing hemoglobin and hematocrit levels [164,165,166], improving kidney function and reducing inflammation [52,167].

In view of the above, future clinical trials should evaluate the safety and efficacy of the concomitant use of SGLT2 inhibitors as a clinical strategy to prevent or better manage IDA associated with incretin receptor agonist-based therapies. Nevertheless, combination therapy with incretin receptor agonists and SGLT2 inhibitors may be considered in patients with IDA related to incretin receptor agonist-based therapies who already have one or more approved indications for SGLT2 inhibitor therapy, namely: T2D with or without established CVD and/or diabetic kidney disease (DKD); heart failure with preserved (HFpEF) or reduced (HFrEF) ejection fraction; CKD at risk of progression [168].

**Figure 1:**
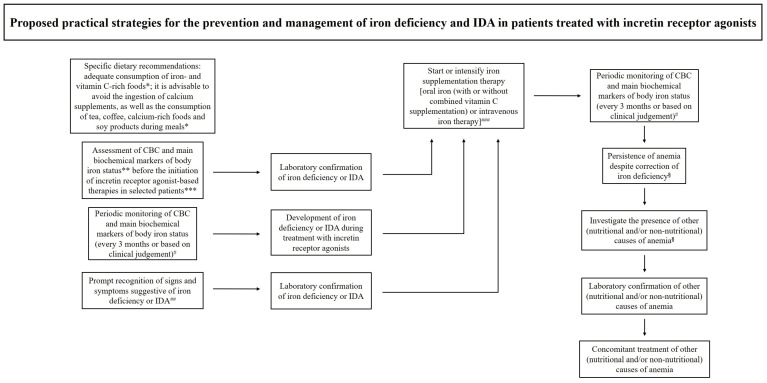
Proposed practical strategies for the prevention and management of iron deficiency and IDA associated with incretin receptor agonist-based therapies. Abbreviations: CBC, complete blood count; IDA, iron deficiency anemia. * Iron-rich foods include lean meat, poultry, fish, shellfish, green leafy vegetables, beans, lentils, peas, nuts, dried fruits, and fortified cereals. Vitamin C-rich foods include citrus fruits, bell peppers, green leafy vegetables, tomatoes, and berries. Ingestion of calcium supplements and consumption of tea, coffee, calcium-rich foods and soy products should be avoided during meals, since these supplements and foods can alter the absorption of non-heme iron. ** CBC should be performed to assess red blood cell (RBC) count, RBC indices (MCV, Mean Corpuscular Volume; MCH, Mean Corpuscular Hemoglobin; MCHC, Mean Corpuscular Hemoglobin Concentration; RDW, Red Blood Cell Distribution Width) and hemoglobin and hematocrit levels; main biochemical markers of body iron status include serum ferritin, transferrin saturation (TSAT), serum iron, serum transferrin, total iron-binding capacity (TIBC), and unsaturated iron-binding capacity (UIBC). Key diagnostic markers of absolute iron deficiency include a serum ferritin level below 30 ng/mL (in the absence of inflammation) and a TSAT value lower than 20%. *** Laboratory testing for the screening and diagnosis of iron deficiency and IDA before the initiation of incretin receptor agonist-based therapies should be considered in selected patients, including patients with signs and symptoms suggestive of iron deficiency or IDA, patients with a previously established diagnosis of iron deficiency or IDA, as well as vulnerable subjects with one or more risk factors for the development or worsening of iron deficiency and IDA. ^#^ CBC and main biochemical markers of body iron status should be regularly assessed (every 3 months) during incretin receptor agonist-based therapies in patients with an established diagnosis of iron deficiency or IDA, as well as in vulnerable subjects with one or more risk factors for the development or worsening of iron deficiency and IDA. However, the frequency of laboratory workup including CBC and main biochemical markers of body iron status in patients treated with incretin receptor agonists can vary (e.g., closer laboratory monitoring) based on clinical judgement, selected circumstances and/or specific factors (e.g., number of risk factors for the development or worsening of iron deficiency and IDA, occurrence of gastrointestinal adverse effects of incretin receptor agonists during incretin receptor agonist dose up-titration, prolonged gastrointestinal adverse effects of incretin receptor agonists, pronounced appetite suppression, rapid weight loss, development of signs and symptoms suggestive of iron deficiency or IDA, moderate-to-severe IDA). In patients prescribed incretin receptor agonists who have an established iron deficiency/IDA, a past history of iron deficiency/IDA, an established anemia other than IDA, or a past history of anemia other than IDA, CBC and main biochemical markers of body iron status may be assessed more frequently. Among patients eligible for incretin receptor agonist-based therapies, low socioeconomic status, female sex, premenopausal status, heavy menstrual bleeding, chronic inflammation, history of bariatric surgery, inadequate iron intake, chronic diseases (including overweight/obesity, type 2 diabetes, chronic kidney disease, heart failure, rheumatic diseases and cancer), malabsorption syndromes (such as inflammatory bowel disease and celiac disease), age > 65 years, use of certain medications (e.g., proton pump inhibitors, histamine H2 receptor antagonists, antacids, aspirin, antiplatelet drugs, non-aspirin nonsteroidal anti-inflammatory drugs, oral anticoagulants), vegetarianism and veganism represent major risk factors for the development or worsening of iron deficiency and IDA. Reassessment of CBC and main biochemical markers of body iron status should also be performed after the initiation of iron supplementation, in order to evaluate the need for ongoing iron administration and to establish the optimal dose, dosing frequency and route of administration of iron supplementation. ^##^ Signs and symptoms suggestive of iron deficiency or IDA (including laboratory findings) include microcytic hypochromic anemia, weakness, fatigue, exercise intolerance, tachycardia, pallor, dyspnea, cold intolerance, koilonychia (spoon-shaped nails), glossitis, cognitive dysfunction, decreased attention and concentration (“brain fog”), mood changes (irritability, depression), lightheadedness, pica [craving non-food substances, such as ice (pagophagia or ice craving)], restless legs syndrome, worsening heart failure, and hair loss. Signs and symptoms suggestive of absolute iron deficiency or IDA (including laboratory findings) should be promptly recognized in patients eligible for incretin receptor agonist-based therapies and in patients who are already receiving incretin receptor agonists, in order to allow appropriate laboratory monitoring and timely intervention. ^###^ Management of iron deficiency or IDA in patients treated with incretin receptor agonists should follow the same principles outlined in the international guidelines for the management of these conditions (particularly in terms of optimal dose, dosing frequency and route of administration of iron supplementation) (DeLoughery et al., 2024—[143]; Iolascon et al., 2024—[144]). Patients receiving oral iron supplementation should not consume coffee, tea, fiber or calcium-containing foods within 1 h before or after taking an iron supplement, since these foods can decrease iron absorption. Intravenous iron therapy may be necessary in the following groups of patients: patients requiring a faster iron replenishment than that achieved with oral iron (e.g., patients with IDA who are scheduled for surgery); patients who have oral iron intolerance; patients in whom oral iron is likely ineffective in the treatment of iron deficiency or IDA (e.g., menstruating women with heavy menstrual bleeding, patients with malabsorption syndromes or impaired iron absorption, patients who underwent bariatric surgery, patients with active inflammatory bowel disease and other chronic inflammatory conditions, patients with chronic gastrointestinal bleeding, patients with ongoing blood loss). ^§^ If patients prescribed incretin receptor agonist-based therapies continue to exhibit anemia despite dietary recommendations, adequate iron supplementation—with or without combined vitamin C supplementation in the case of oral iron supplementation—and correction of iron deficiency, the presence of other nutritional and/or non-nutritional causes of anemia should be sought and treated. Other types of nutritional anemia are those related to the deficiency of one or more of the following micronutrients: vitamin B1 (thiamine), vitamin B2 (riboflavin), vitamin B6 (pyridoxine), folic acid (vitamin B9), cobalamin (vitamin B12), vitamin A, vitamin C, and copper.

Figure 1 illustrates the proposed practical strategies for the prevention and management of iron deficiency and IDA associated with incretin receptor agonist-based therapies.

## 5. Concluding Remarks and Future Perspectives

Besides the well-established benefits of incretin receptor agonists in terms of weight loss, glucose control and cardiorenal protection in patients with overweight/obesity and T2D, preliminary evidence shows that the use of these drugs can be associated with the development of micronutrient deficiencies and nutritional complications, including iron deficiency and IDA. Preventing or correcting iron deficiency in patients treated with incretin receptor agonists is crucial to improve patient outcomes and reduce the risk of adverse consequences of impaired iron metabolism, including IDA.

The likelihood of developing iron deficiency and IDA may be higher in patients treated with incretin receptor agonists, particularly in the presence of specific risk factors, such as low socioeconomic status, female sex, premenopausal status, heavy menstrual bleeding, chronic inflammation, history of bariatric surgery, inadequate iron intake, chronic diseases, malabsorption syndromes, use of certain medications, vegetarianism and veganism. Therefore, routine nutritional screening (before and after the initiation of incretin receptor agonist-based therapies), prompt identification of nutritional deficiencies (including iron deficiency) and the involvement of registered dietitian nutritionists represent key elements in the management of patients receiving incretin receptor agonist-based therapies. Indeed, patients treated with incretin receptor agonists require a precision nutrition approach based on their specific nutritional needs. According to this precision nutrition approach, patients treated with incretin receptor agonists should consume adequate amounts of nutrient-dense (minimally processed) foods, lean proteins, iron-rich foods and vitamin C-rich foods to promote healthy weight loss (fat mass loss with preservation of lean mass) and to prevent or correct iron deficiency and IDA.

We acknowledge that the current evidence regarding the potential relationship between incretin receptor agonist-based therapies, iron deficiency and anemia remains limited and confined to a few retrospective observational studies [28,29], with the prevalence of IDA reported to be as low as 3.2% after 12 months of GLP-1 RA therapy [28]. The prevalence of IDA associated with incretin receptor agonist-based therapies appears to be relatively low [28] compared with the well-established clinical benefits of these therapies across a broad spectrum of chronic conditions [2,169]. Therefore, the prescription of incretin receptor agonists should not be unjustifiably restricted by the possible and modest risk of iron deficiency and IDA in patients with one or more approved indications for therapeutic use of these agents. Although iron deficiency and IDA currently appear to be uncommon adverse effects of incretin receptor agonist-based therapies, clinicians should be aware of the possibility of their occurrence to ensure appropriate prevention and management of these nutritional complications. Future large-scale, prospective studies are certainly warranted to better establish the long-term effects of incretin receptor agonists on iron homeostasis and the causal relationship between the initiation of incretin receptor agonist-based therapies and the development of iron deficiency and IDA. Additionally, future prospective studies should investigate whether incretin receptor agonist dose is correlated with the risk of iron deficiency/IDA and whether selective GLP-1 RA and dual GIP/GLP-1 RA exert differential effects on iron homeostasis. Future mechanistic studies are also needed to clearly establish the exact mechanisms underlying the potential development of iron deficiency and IDA in patients treated with incretin receptor agonists, although current preliminary evidence mainly points to inadequate dietary iron intake and decreased intestinal iron absorption. These studies would also help clarify the optimal dose, dosing frequency and route of administration of iron supplementation in patients treated with incretin receptor agonists who develop iron deficiency and IDA. Moreover, future prospective studies should aim to identify potential predictive biomarkers of susceptibility to the development of iron deficiency and IDA prior to the initiation of incretin receptor agonist-based therapies (predictive biomarkers), as well as specific strategies for the prevention and therapeutic management of this nutritional complication (particularly in vulnerable subjects with risk factors for the development or worsening of iron deficiency and IDA). In this regard, incretin receptor agonists should not be regarded as agents that inevitably cause inadequate dietary iron intake and/or decreased intestinal iron absorption. Conversely, it is likely that these agents contribute to inadequate dietary iron intake and/or decreased intestinal iron absorption only in a minority of vulnerable subjects carrying specific risk factors that remain to be better characterized.

We acknowledge that the strategies proposed in the present manuscript for the prevention and management of incretin receptor agonist-associated iron deficiency/IDA should only be regarded as practical clinical approaches deriving from the existing recommendations for the prevention and management of iron deficiency and IDA [143,144]. The cost-effectiveness of the strategies proposed herein for the prevention and management of incretin receptor agonist-associated iron deficiency/IDA should be appropriately assessed in future clinical trials. Meanwhile, assessment of CBC and main biochemical markers of body iron status should be performed before the initiation of incretin receptor agonist-based therapies in patients with signs and symptoms suggestive of iron deficiency or IDA, in patients with a previously established diagnosis of iron deficiency or IDA, as well as in vulnerable subjects with one or more risk factors for the development or worsening of iron deficiency and IDA. Subsequently, CBC and main biochemical markers of body iron status should be regularly assessed during incretin receptor agonist-based therapies in patients with established iron deficiency or IDA, as well as in vulnerable subjects with one or more risk factors for the development or worsening of iron deficiency and IDA, with the frequency of laboratory workup being determined by clinical judgement, selected circumstances and/or specific factors (e.g., number of risk factors for the development or worsening of iron deficiency and IDA, occurrence of gastrointestinal adverse effects of incretin receptor agonists during incretin receptor agonist dose up-titration, prolonged gastrointestinal adverse effects of incretin receptor agonists, pronounced appetite suppression, rapid weight loss, development of signs and symptoms suggestive of iron deficiency or IDA, moderate-to-severe IDA). Future studies are warranted to determine the most appropriate frequency for CBC monitoring and assessment of main biochemical markers of body iron status in different subgroups of patients treated with incretin receptor agonists.

Among patients who are prescribed incretin receptor agonists, iron supplementation (with or without combined vitamin C supplementation) should be started in individuals with an established diagnosis of iron deficiency or IDA (despite the recommended adequate consumption of foods high in iron and vitamin C) before the initiation of incretin receptor agonist-based therapies, or in patients who develop iron deficiency or IDA after the initiation of incretin receptor agonist-based therapies (despite the recommended adequate consumption of foods high in iron and vitamin C). If patients prescribed incretin receptor agonist-based therapies continue to exhibit anemia despite dietary recommendations, adequate iron supplementation—with or without combined vitamin C supplementation in the case of oral iron supplementation—and correction of iron deficiency, the presence of other nutritional and/or non-nutritional causes of anemia should be sought and treated.

Until further data from large population studies become available, clinical awareness and individualized nutritional assessment may be appropriate for the prevention and management of iron deficiency and IDA in patients receiving incretin receptor agonist-based therapies, particularly in those with one or more risk factors for the development or worsening of these nutritional complications.

## Figures and Tables

**Table 1:** Potential mechanisms underlying the development of iron deficiency and IDA (beyond classic risk factors for iron deficiency and IDA) in patients treated with incretin receptor agonists.

Inadequate dietary iron intake Potential causes: Reduction in food intake (reduced micronutrient and macronutrient intake) caused by incretin receptor agonists (decreased appetite and increased satiety); gastrointestinal adverse effects (especially nausea, dyspepsia, vomiting and diarrhea) of incretin receptor agonists, which further contribute to lower energy and nutrient intake (fewer, smaller and less varied meals) due to changes in habitual eating patterns and avoidance of foods that exacerbate these adverse effects.
Low dietary variety, monotonous diets and changes in food preferences, with reduction in the consumption of iron-rich foods (particularly meat, fish and seafood)
Impairment of intestinal iron absorption Potential causes: Delayed gastric emptying and reduced small intestinal motility (caused by incretin receptor agonists), resulting in altered timing and efficiency of nutrient delivery to absorption sites in the small intestine; decreased gastric acid secretion (caused by incretin receptor agonists).
Vitamin B2 (riboflavin) deficiency *, which may result in reduced iron absorption, increased gastrointestinal iron losses and/or impaired iron mobilization from iron stores Potential causes: Reduced micronutrient (vitamin B2) intake and/or impaired intestinal micronutrient (vitamin B2) absorption related to the use of incretin receptor agonists.
Potential ability of incretin receptor agonists to modify gut microbiota composition *, resulting in altered iron homeostasis

* These mechanisms are hypothetical and require confirmation through mechanistic studies.

## Data Availability

Not applicable.
